# Human CD49a^+^ Lung Natural Killer Cell Cytotoxicity in Response to Influenza A Virus

**DOI:** 10.3389/fimmu.2018.01671

**Published:** 2018-07-20

**Authors:** Grace E. Cooper, Kristoffer Ostridge, Salim I. Khakoo, Tom M. A. Wilkinson, Karl J. Staples

**Affiliations:** ^1^Clinical and Experimental Sciences, Faculty of Medicine, Sir Henry Wellcome Laboratories, Southampton General Hospital, University of Southampton, Southampton, United Kingdom; ^2^Southampton NIHR Respiratory Biomedical Research Unit, Southampton General Hospital, Southampton, United Kingdom; ^3^Wessex Investigational Sciences Hub, Faculty of Medicine, Southampton General Hospital, University of Southampton, Southampton, United Kingdom

**Keywords:** natural killer cells, human lung, viral infection, cytotoxicity, CD49a

## Abstract

Influenza A virus (IAV) is a major global public health burden due to its routine evasion of immunization strategies. Natural killer (NK) cells are innate cytotoxic cells with important antiviral activity in the human body, yet the function of these cells in the control of IAV infection is unclear. The aim of this study was to determine the role of lung NK cell cytotoxic responses to IAV. Human lung explants were infected *ex vivo* with IAV, and lung NK cell activation was analyzed by flow cytometry. Cytotoxic responses of NK cell subsets against IAV-infected macrophages were measured by flow cytometry and ELISA. Despite reports of hypofunctionality in the pulmonary environment, human lung-associated NK cells responded rapidly to *ex vivo* IAV infection, with upregulation of surface CD107a 24 h post-infection. The lung NK cell phenotype is similar in maturity and differentiation to NK cells of the peripheral blood but a unique CD56^bright^CD49a^+^CD103^+^CD69^+^ NK cell population was identified in the lung, indicating NK cell residency within this organ. In response to *ex vivo* IAV infection a greater proportion of resident CD56^bright^CD49a^+^ NK cells expressed surface CD107a compared with CD56^bright^CD49a^−^ NK cells, suggesting a hyperfunctional NK cell population may be present within human lung tissue and could be the result of innate immunological training. Furthermore, NK cells provided significant antiviral, cytotoxic activity following contact with influenza-infected cells, including the production and release of IFN-γ and granzyme-B resulting in macrophage cell death. These results suggest that a resident, trained NK cell population are present in the human lung and may provide early and important control of viral infection. A greater understanding of this resident mucosal population may provide further insight into the role of these cells in controlling viral infection and generating appropriate adaptive immunity to IAV.

## Introduction

Influenza A virus (IAV) is a single-stranded ribonucleic acid virus of the *Orthomyxoviridae* family causing acute infection of the upper and lower respiratory tract (1). IAV infection remains a global public health burden with 3–5 million cases of severe illness and 500,000 deaths worldwide, annually (2). Vaccination is currently the best method of controlling viral transmission; however, annual influenza vaccines are limited in efficacy due to rapid viral evolution, time required for production and ineffectiveness in high-risk groups (3, 4). Improving the current immunization strategies requires a more advanced understanding of both innate and adaptive human immunity to influenza virus (5).

The lungs are one of the largest reservoirs of natural killer (NK) cells in the body, yet the function of these cells in pulmonary viral infection is poorly understood (6, 7). NK cells are antiviral lymphocytes essential to the control of human pathogens such as hepatitis C virus, cytomegalovirus, and human immunodeficiency virus (8, 9). NK cells aid viral clearance through secretion of pro-inflammatory cytokines such as IFN-γ as well as cytotoxic granules and engagement of death receptors, which stimulate target cell apoptosis (8). Different subpopulations of human NK cells can be identified through high and low expression of CD56, and these populations of NK cells have been ascribed different functions. CD56^bright^ NK cells are thought to be predominantly cytokine producing while CD56^dim^ NK cells represent the canonical cytotoxic NK cell; however, these functional outputs appear dependent on the type of *in vitro* stimulation and the role of these NK cell subtypes within the human body remain largely unexplored (10–14).

Natural killer cells recognize virally infected cells through the integration of signaling from activatory and inhibitory germline encoded receptors on the NK cell surface (15). *In vitro* binding studies have shown that the activatory NK cell receptors NKG2D and NKp46, and inhibitory KIR2DL2 NK cell receptor bind influenza-infected cells (16–18). Furthermore, strong IFN-γ responses are observed in the NK cells of the peripheral blood following influenza vaccination (19–22). The majority of mouse models of influenza infection implicate a protective role for NK cells during infection (23–27). Indeed *Ncr1*^−/−^ (NKp46) knockout in the mouse results in lethal influenza infection (24). However, in most high dose infection models, murine NK cells appear to play a detrimental role, contributing to influenza pathogenesis (28, 29). This may suggest there is a delicate balance between protective and destructive NK cell activation during IAV infection. But due to differences in influenza strain, dose, and genetic background of the mice, it is difficult to delineate what role NK cells may play in murine models of influenza infection.

The majority of studies investigating the human NK cell responses to influenza utilize peripheral blood NK cells (17, 19–22). However, organ-resident NK cell populations have recently been identified in the liver, uterus, and secondary lymphoid tissue, with resident populations expressing CD103, CD69, and CD49a (30–34). Phenotypically different from the blood, these populations have been linked to NK cell memory and may possess different functionality as a result of “training” by their environment (35). Lung NK cell residency has been disputed, with lung NK cells often described as hypofunctional (36–39). However, there is some evidence that NK cell memory can be generated to respiratory virus, as murine influenza infection induced liver CD49a^+^ NK cells that are protective against influenza infection (40). Furthermore, influenza vaccination results in a more potent NK cell response to influenza in human peripheral blood mononuclear cells (PBMCs) (41).

The contribution of NK cells to pulmonary health and disease is poorly understood in humans and how NK cells may respond to influenza infection has not been characterized in a model effectively recapitulating human infection. The aim of this study was therefore to investigate the role of human lung NK cells in early influenza control using our previously characterized lung explant model of influenza infection (42–44).

## Materials and Methods

### Volunteer Recruitment

Lung tissue distal from tumor sites and peripheral blood was obtained from lobectomy patients. Blood was also obtained from healthy human volunteers. All studies were approved by Southampton and South West Hampshire Research Ethics Committees (13/SC/0416 for healthy control group, 09/H0504/109 for resection and blood donors, and 15/SC/0528 for age comparison). All participants provided written informed consent.

### Preparation of Lung Tissue and Explant Infection

Lung tissue explants were prepared as previously described and infected with X31 (H3N2) IAV (Virapur) as previously described (43, 44). Briefly, lung tissue explants were cut into 4–6 mm^2^ pieces with 6 fragments/well and washed thoroughly with cold RPMI. The lung explants were then rested for 16 h in complete RPMI [RPMI with 10% FCS, 2 mg/mL l-glutamine, 0.05 IU/mL penicillin, 50 µg/mL streptomycin, and 0.25 µg/mL amphotericin B (Sigma)] to remove contaminant blood. For NK cell phenotyping, the lung tissue was digested in 0.5 mg/mL collagenase, filtered through a 0.7-µm filter, and mononuclear cells isolated by density gradient centrifugation over a Ficoll-paque layer. Cells isolated from the buffy coat were then stained for flow cytometry. For IAV infection of lung explants, lung fragments were washed in PBS and infected with 200,000 pfu/mL live or UV-irradiated X31 (H3N2) IAV (Virapur) following rest (42, 45). UV-irradiated X31 was created by exposing live X31 to UV light for 2 h on ice. 2 hpi extracellular virus was removed by washing in PBS and explants cultured in fresh media for a further 22 h, or for stated time point. X31 replication was detected through intracellular viral nucleoprotein-1 (NP1) expression. In addition, lung explants were alternatively treated with phorbol myristate acetate/ionomycin (PMA/I—1.34 µM and 81 nM, respectively). After infection NK cells were dispersed from tissue using 0.5 mg/mL collagenase, the digest filtered and cells stained for flow cytometry.

A median of 410,676 ± 153,226 CD45^+^ leukocytes were isolated per resection sample, according to flow cytometry analysis. The median total events in the NK cell gate was 10,011 ± 9,073 and a minimum value of 2,760 (*N* = 8). For the rarer CD56^bright^ CD49a^+^ NK cells, the median number of events was 940 ± 194 and a minimum value of 130; a cutoff of a minimum 100 events in this gate was required to be included in the analysis (*N* = 8). The same cutoff was used in the functional studies of the CD56^bright^CD49a^+^ population. The median number of events for CD56^bright^CD49a^+^ NK cells in the X31-infected explant was 1,035± and a minimum value of 164 (*N* = 5). This was also typical of the untreated and UV-irradiated X31 treated explant tissue.

### Blood NK Cell and Monocyte Isolation and Differentiation

Human PBMCs were isolated from heparinized blood of healthy volunteers or from cancer resection donors. Blood was diluted 1:1 with PBS and mononuclear cells isolated by density gradient centrifugation on Ficoll-Paque (GE Healthcare). Monocytes were isolated from PBMC by CD14-positive magnetic-activated cell sorting (MACS) (Miltenyi Biotec). Monocytes were seeded at 500,000 cells/well (1 × 10^6^ cells/ml) and differentiated into monocyte-derived macrophages (MDMs) over 12 days in complete RMPI with 2 ng/mL GM-CSF, as described previously (46). Autologous NK cells were isolated from the CD14-depleted MACS filtrate by negative selection with an NK cell isolation kit (Miltenyi) according to the manufacturer’s instructions. Purified NK cells (94.28% CD3^−^CD56^+^, *N* = 5) were frozen in FCS containing 10% DMSO (Sigma) until use.

### Macrophage Infection and Co-Culture With NK Cells

Monocyte-derived macrophage infection was performed as previously described (43). MDMs were treated with 500 pfu/mL of live or UV-irradiated X31 virus for 2 h, before removal of extracellular virus by washing with PBS and incubation for a further 22 h. UV-irradiated X31 was created by exposing live X31 to UV light for 2 h on ice. After 24 h, autologous NK cells were thawed and cultured with untreated or infected MDMs for 6 h at an effector:target (E:T) of 1:5. This E:T was chosen following testing of a range of E:T of NK cells to X31-infected MDMs, as shown in Figure S1 in Supplementary Material and was chosen for the optimal detection of NK cell IFN-γ production. NK cell degranulation, IFN-γ and GzmB production and MDM viability were measured by flow cytometry. Adherent MDMs were collected for flow cytometry with non-enzymatic cell dissociation solution (Sigma). To measure the accumulation of intracellular molecules 2 µM Monensin (eBiosciences) was added into the co-culture 1 h after the addition of NK cells. Physical contact between MDMs and NK cells was prevented through culturing in a transwell system with NK cells in the top compartment (Costar). HLA class I binding was blocked through incubation of 20 µg/mL αHLA-A/B/C (W6/32; BioLegend) with MDM 20 min prior to addition of NK cells.

### Flow Cytometry

Flow cytometry was performed as previously described, all steps were performed at 4°C for 30 min, unless otherwise stated (43). Cell viability was assessed by staining with Zombie Violet Fixable Viability Kit (BioLegend) in PBS. Surface marker staining was performed in 2 mM EDTA and 0.5% bovine serum albumin (FACS buffer) with 2 mg/mL human IgG (Sigma). Cellular surface markers were stained with the following antibodies; 5 µL αCD3-PerCP (UCHT1), 5 µL αCD56-PECy7 (HCD56), 5 µL αCD57-PacificBlue (HCD57), 5 µL αCD103-APC (Ber-ACT8), 5 µL αCD69-BV421 (FN50), 5 µL αCD107a-BV510 (H4A3) (BioLegend), 5 µL αCD45-BV510 (HI30), 5 µL αCD16-FITC (3G8), 20 µL αCD49a-PE (SR84), 20 µL αCD158b-PE (CH-L) (BD Biosciences) and with corresponding isotype controls; Mouse IgG1-PerCP, IgG1-PECy7, IgG2b-PE, IgM-Pacific Blue, IgG1-APC, IgG1-BV421 (BioLegend) Mouse IgG1-BV510, IgG1-FITC, IgG1-PE (BD Biosciences). Macrophages and epithelia were stained with, 5 µL αHLA-DR-APC-Cy7 (L243) and 20 µL αEpCAM-PerCP-Cy5.5 (EBA-1), 5 µL αHLA-ABC-PE (W6/32) (BD Biosciences). Corresponding isotype controls not previously stated included mouse IgG2a-APC-Cy7 (BD Biosciences) and IgG2a-PE (Life Tech). Cells were then fixed and permeabilized with Cytofix/Cytoperm (BD Biosciences, Oxford, UK) for 20 min before intracellular staining. Intracellular staining was performed in 1× Permwash (BD Biosciences) in FACS staining buffer. Intracellular markers were assessed with 2 µL α-NP-1-FITC (ab20921) (Abcam), 5 µL αIFN-γ-PerCP-Cy5.5 (4S.B3), or 5 µL αGzm-B-APC (QA16A02) (BioLegend). Flow cytometric analysis was performed on a FACSAria using FACSDiva software v5.0.3 (BD Biosciences) and FlowJo v10 (Tree Star). The t-SNE algorithm was applied within FlowJo v10 for 1,000 iterations to produce 2D projections of the data using a perplexity value of 20. FCS files from the lung and matched blood, or lung only, were downsampled to 300 or 600 cells, respectively.

### ELISA

IFN-γ ELISA MAX (BioLegend), and Granzyme B duoset ELISA (R&D Systems) were all carried out according to the manufacturer’s protocol.

### Statistics

Statistical analyses were performed using either a Chi-squared test, Fisher’s test, Wilcoxon’s matched-pairs signed-rank test, Mann–Whitney *U* test, Kruskal–Wallis or Friedman test with Dunn’s multiple comparison testing as appropriate (GraphPad Prism v7.0, GraphPad Software Inc., San Diego, CA, USA). Data are expressed as medians. Results were considered significant if *P* < 0.05.

## Results

### NK Cells Are Present at High Frequencies in Human Lung Parenchyma

**Figure 1:**
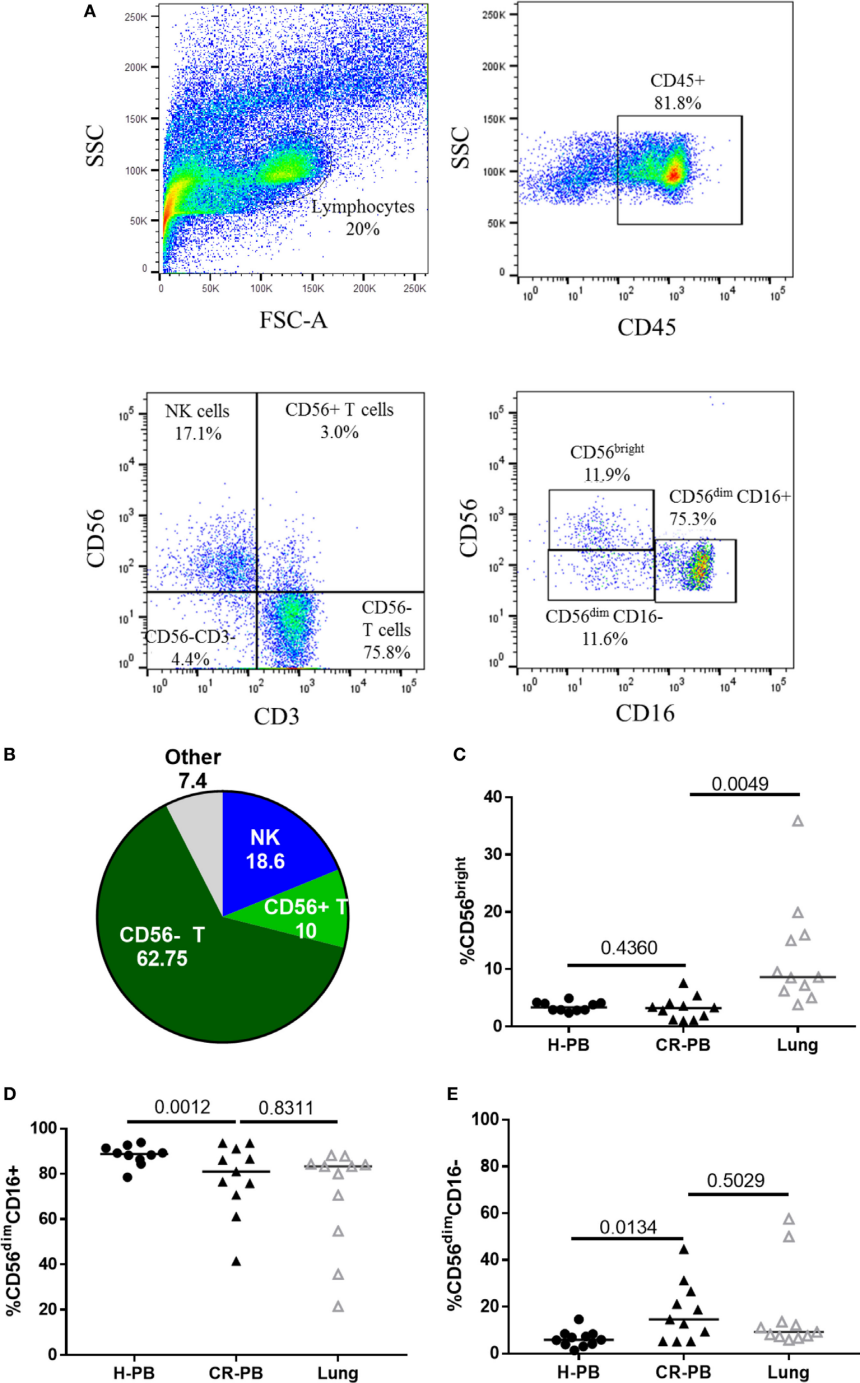
Lung-associated natural killer (NK) cells are highly differentiated and similar to the peripheral blood of healthy donors [healthy peripheral blood (H-PB)] and those undergoing cancer resection [peripheral blood from cancer resection donor (CR-PB)]. **(A)** Representative gating strategy to define NK cells and NK cell subpopulations isolated from human lung parenchyma. **(B)** Quantification of lung CD45^+^ lymphocytes, *N* = 23. **(C–E)** Proportions of CD56^bright^, CD56^dim^CD16^+^, and CD56^dim^CD16^−^ NK cells in H-PB (*N* = 14), lung tissue (*N* = 23), and matched blood (CR-PB, *N* = 23).

Natural killer cells were isolated from human lung parenchyma, matched peripheral blood and peripheral blood from healthy controls. NK cells were defined as CD45^+^CD3^−^CD56^+^ cells by flow cytometry and made up a median of 18.55 ± 14.98% of CD45^+^ lymphocytes isolated from the human lung parenchyma (*N* = 17, Figure 1). CD56^bright^CD16^−^, CD56^dim^CD16^+^, and CD56^dim^CD16^−^ NK cells were all identified in lung and blood with CD56^dim^CD16^+^ and CD56^dim^CD16^−^ found in similar proportions between the lungs and peripheral blood (*P* = 0.83 and *P* = 0.50, Figures 1C–E). As described by Marquardt et al., the majority of lung-associated NK cells are canonical cytotoxic CD56^dim^CD16^+^ cells (39). However, we observed an increased proportion of CD56^bright^ NK cells in the lungs compared with matched peripheral blood (8.6 vs 3.3%, *P* = 0.0049, Figure 1C). Furthermore, fewer CD56^dim^CD16^+^ NK cells (*P* = 0.0012) and a greater proportion of CD56^dim^CD16^−^ NK cells were isolated from the blood of cancer resection patients compared with healthy control blood.

**Figure 2:**
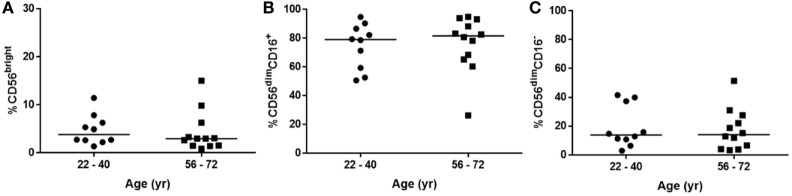
Natural killer cell subpopulation proportions are not affected by age. **(A)** CD56^bright^, **(B)** CD56^dim^CD16^+^, and **(C)** CD56^dim^CD16^−^ proportions in peripheral blood of healthy donors aged 22–40 (*N* = 10) and 56–72 (*N* = 12). Statistical analysis by Mann–Whitney *U* test, lines describe medians.

These differences in the NK cell subpopulations may reflect differences in the cohort age and smoking status between healthy controls and donors undergoing resection surgery, as shown in Table 1. To assess the effect of age on NK cell subpopulations of the blood, the proportions of CD56^bright^ and CD56^dim^ NK cells were analyzed from the blood of healthy controls aged above and below 40 (donor demographics shown in Table 2). However, no differences were observed in the peripheral NK cell subpopulations of these two cohorts (Figure 2).

**Table 1:** Cohort demographics for resection donors and healthy controls.

	Cancer resection donors	Healthy control donors (phenotyping)	*P*-value
Number of patients	35	15	
Age (years)	70 *(9.75)*	24 *(8)*	<0.0001^b^
M/F	21/14	10/5	0.7570^c^
Smoking status, never/ex/current/unknown	5/22/7/1	10/4/1	0.0013^d^
Pack-years of smoking	40 *(33.75)*	NA	NA
FEV1%	86 *(28.5)*	NA	NA
FEV1/FVC ratio	0.65 *(0.15)*	NA	NA
Resection location, LUL/LLL/RUL/RML/RLL	8/7/11/3/3	NA	NA
	1 RUL + LUL^a^		
	1 RUL + RML^a^		
	1 Left pneumonectomy^a^		

*Median values and italicized interquartile range are shown*.

*NA, data not available*.

*^a^Additional locations of resection surgeries*.

*^b^Two-tailed Mann–Whitney test*.

*^c^Fisher’s test*.

*^d^Chi-square test*.

**Table 2:** Cohort demographics for age comparison study.

	Healthy control (under 40)	Healthy control (over 40)	*P*-value
Number of patients	10	12	
Age (years)	34 *(5.5)*	66.5 *(7)*	<0.0001^a^
M/F	4/6	4/8	0.5315^b^
Smoking status, never/ex/current	6/3/1	6/6/0	0.3998^c^
Pack-years of smoking	0 *(1.605)*	2 *(20)*	0.2332^a^
FEV1%	97 *(17.75)*	108 *(16.5)*	0.0143^a^
FEV1/FVC ratio	0.84 *(8.5)*	0.78 *(2.75)*	0.1945^a^

*Median values and italicized interquartile range are shown*.

*^a^Two-tailed Mann–Whitney test*.

*^b^Fisher’s test*.

*^c^Chi-squared test*.

**Figure 3:**
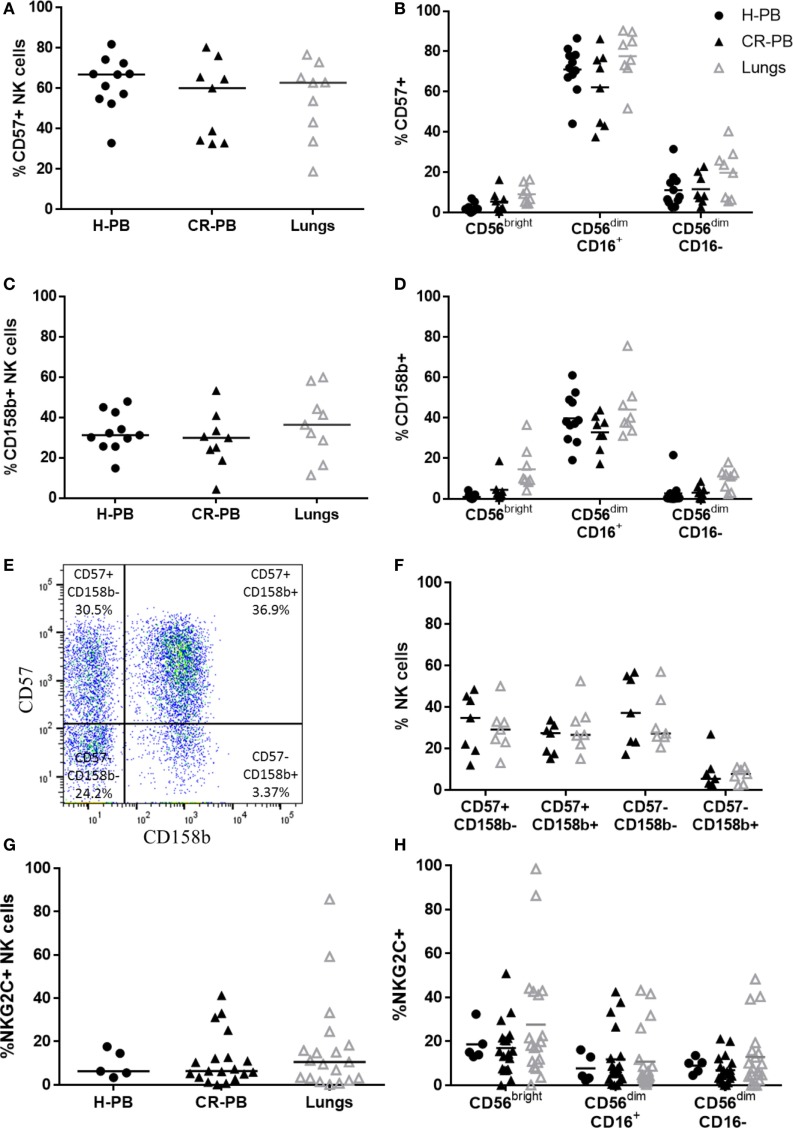
CD57, CD158b, and NKG2C expression on lung and blood natural killer (NK) cells. CD57 **(A)** and CD158b **(C)** expression on NK cell subsets in peripheral blood of healthy controls [healthy peripheral blood (H-PB) *N* = 11] and peripheral blood [peripheral blood from cancer resection donor (CR-PB)] and lung of cancer resection patients (*N* = 9). Lines describe medians, comparison between healthy and resection blood by Mann–Whitney *U* test and between lung and CR-PB by Wilcoxon signed-rank test. **(B,D)** CD57 and CD158b expression on CD56^bright^, CD56^dim^CD16^+^, and CD56^dim^CD16^−^ NK cells from the blood and lung. Statistical analysis of H-PB to CR-PB by Kruskal–Wallis test with Dunn’s multiple comparison correction and CR-PB to lung tissue by Friedman’s test with Dunn’s multiple comparison correction. **(E)** Representative flow cytometry plot describing lung NK cell CD57 and CD158b expression. **(F)** CD57 and CD158b expression on NK cells from the lung and matched blood [CR-PB (*N* = 7)]. Statistical analysis by Friedman’s test with Dunn’s multiple comparison correction. **(G)** NKG2C expression on healthy controls (*N* = 5), cancer resection donor blood and lung tissue (*N* = 19). **(H)** NKG2C expression on CD56^bright^, CD56^dim^CD16^+^, and CD56^dim^CD16^−^ NK cells from the blood and lung.

To evaluate the maturity of lung NK cells, the expression of CD57 and CD158b (KIR2DL2/L3/S2) was analyzed on lung and blood NK cells. CD57 is expressed in the late stages of NK cell differentiation and is associated with increased NK cell functionality (47, 48). KIR expression also increases during NK cell maturation, as NK cells gain cytotoxic function (49, 50). Although CD158b does not evaluate the expression of all KIR, which vary across individuals, it represents KIR from both haplotypes A and B (51). Individuals with haplotype A typically possess KIR alleles with a more inhibitory role than the KIR haplotype B, which has a more activating effect on NK cell function (52). Both CD57 and CD158b were expressed equivalently between lung and matched peripheral blood (Figures 3A,C, *P* = 0.91 and *P* = 0.07, respectively). Furthermore, no significant differences were observed in the expression of either CD57 or CD158b on CD56^bright^ or CD56^dim^ NK cell subpopulations (Figures 3B,D). To investigate whether the differentiation state of CD158b-positive and -negative NK cells were different between matched blood and lung, the co-expression of CD57 and CD158b was analyzed, as shown in Figures 3E,F; however, no significant differences were observed [*P* > 0.9999 for each analysis of CD57^+^CD158b^−^, CD57^−^CD158b^+^, double-positive (DP), and double-negative NK cells]. In addition, NKG2C, an activating receptor associated with CMV immunity and memory response was not found to be differentially expressed between blood and lung (Figures 3G,H) (53, 54). Thus, lung NK cells appear to mirror the phenotype of the peripheral blood, as highly mature and terminally differentiated NK cells. These results confirm reports by Marquardt et al. and other studies from the human lung (39, 55).

### Distinct CD49a^+^CD103^+^CD69^+^ NK Cell Populations Are Present in the Lung Parenchyma

**Figure 4:**
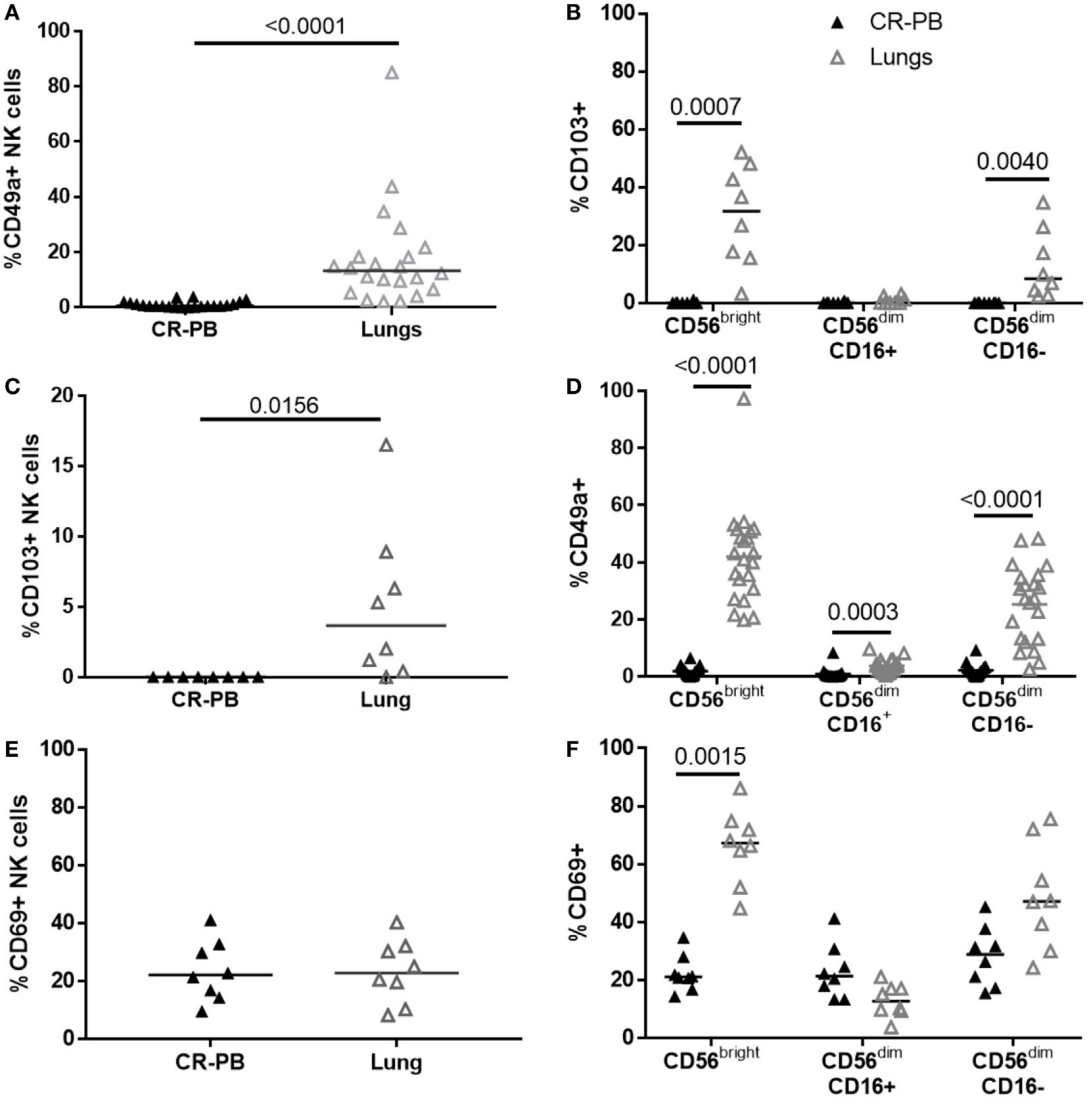
Distinct natural killer (NK) cell populations are present in the blood, but not the lung. **(A,C,E)** Flow cytometric analysis of CD49a (*N* = 22), CD103 (*N* = 8), and CD69 (*N* = 8) expression on lung and blood NK cells. Statistical analysis performed by Wilcoxon signed-rank test. Lines describe medians. **(B,D,F)** Residency marker expression on CD56^bright^ and CD56^dim^ NK cell subsets. Statistical analysis by Friedman’s test with Dunn’s multiple comparison correction.

Natural killer cells isolated from the lungs and peripheral blood appear phenotypically similar in terms of their CD16, CD57, CD158b, and NKG2C expression (Figures 1 and 3); however, a distinct CD49a^+^ NK cell population was identified from the lung parenchyma, which was not found in the circulation (Figure 4). CD49a^+^ NK cells made up 13.3 ± 11% of the total lung-associated NK cell population and were primarily CD56^bright^CD16^−^ and CD56^dim^CD16^−^ NK cells (42.2 and 27.3%, respectively, Figures 4A,B). Negligible amounts of CD49a were detected on CD56^dim^CD16^+^ NK cells. Other putative markers of residency including CD69 and CD103 were also identified in the lung parenchyma with 3.65 ± 7.65% of lung NK cells expressing CD103^+^, a marker also not found in the blood (*P* = 0.016, Figures 4C,D). The expression of CD103 mirrored that of CD49a, with most CD103 expressed on CD56^bright^ and CD56^dim^CD16^−^ NK cells. Although CD69 was not expressed differently between blood and lung at the whole NK cell level (*P* = 0.85), CD56^bright^ lung NK cells expressed greater levels of CD69 compared with the blood (*P* = 0.0015, Figures 4E,F). CD69 was expressed at similar levels by lung CD56^dim^CD16^+^ and CD56^dim^CD16^−^ NK cells (*P* = 0.33 and 0.18).

**Figure 5:**
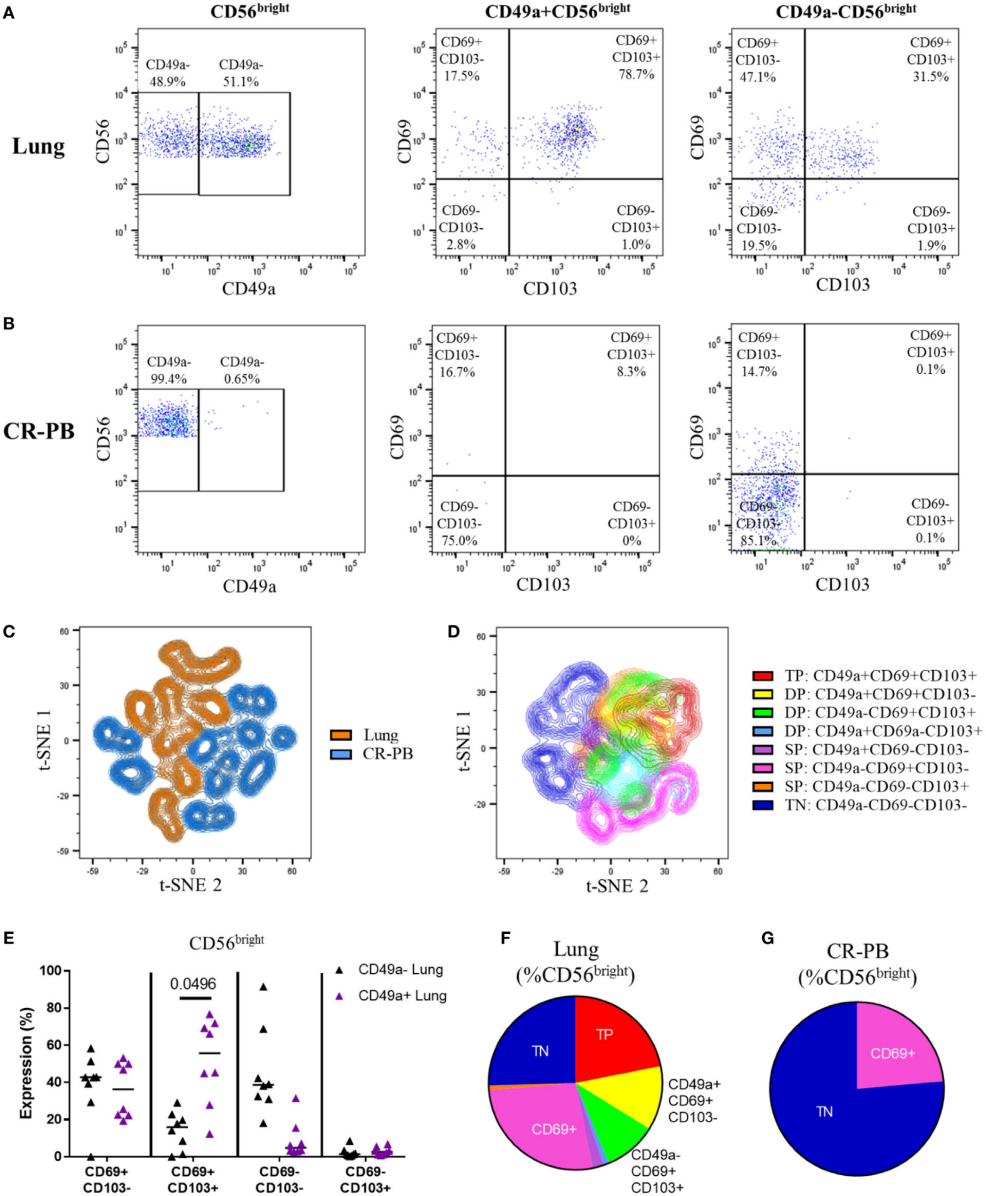
CD56^bright^ CD49a^+^ lung natural killer (NK) cells co-express CD69 and CD103. **(A,B)** Gating strategy to define co-expression of CD69 and CD103 on CD49a^+^ and CD49a^−^ populations of CD56^bright^ NK cells isolated from the lung **(A)** and blood [peripheral blood from cancer resection donor (CR-PB)] **(B)**. **(C)** t-SNE plot of CD56^bright^ NK cells in matched blood (blue) and lung (orange) based on CD49a, CD69, and CD103 expression (*N* = 16, 8 individuals). From each sample, 300 events were randomly selected. Perplexity = 20, 1,000 iterations. **(D)** t-SNE plot of CD56^bright^ NK cells isolated from the lungs based on CD49a, CD69, and CD103 expression (*N* = 8). Cells are colored according to the expression of each marker, showing single-positive (SP), double-positive (DP), and triple-positive (TP) populations. From each sample, 600 events were randomly selected. Perplexity = 20, 1,000 Iterations. **(E)** Quantification of CD69 and CD103 expression of CD49a^+^ and CD49a^−^CD56^bright^ NK cell populations of the lung and blood. Lines described medians, statistical analysis by Friedman’s test with Dun’s correction (*N* = 8). Overall *P*-value <0.001. **(F,G)** Quantification of residency marker expression on CD56^bright^ NK cells of matched blood **(F)** and lung **(G)**. Key as shown in panel **(D)**.

**Figure 6:**
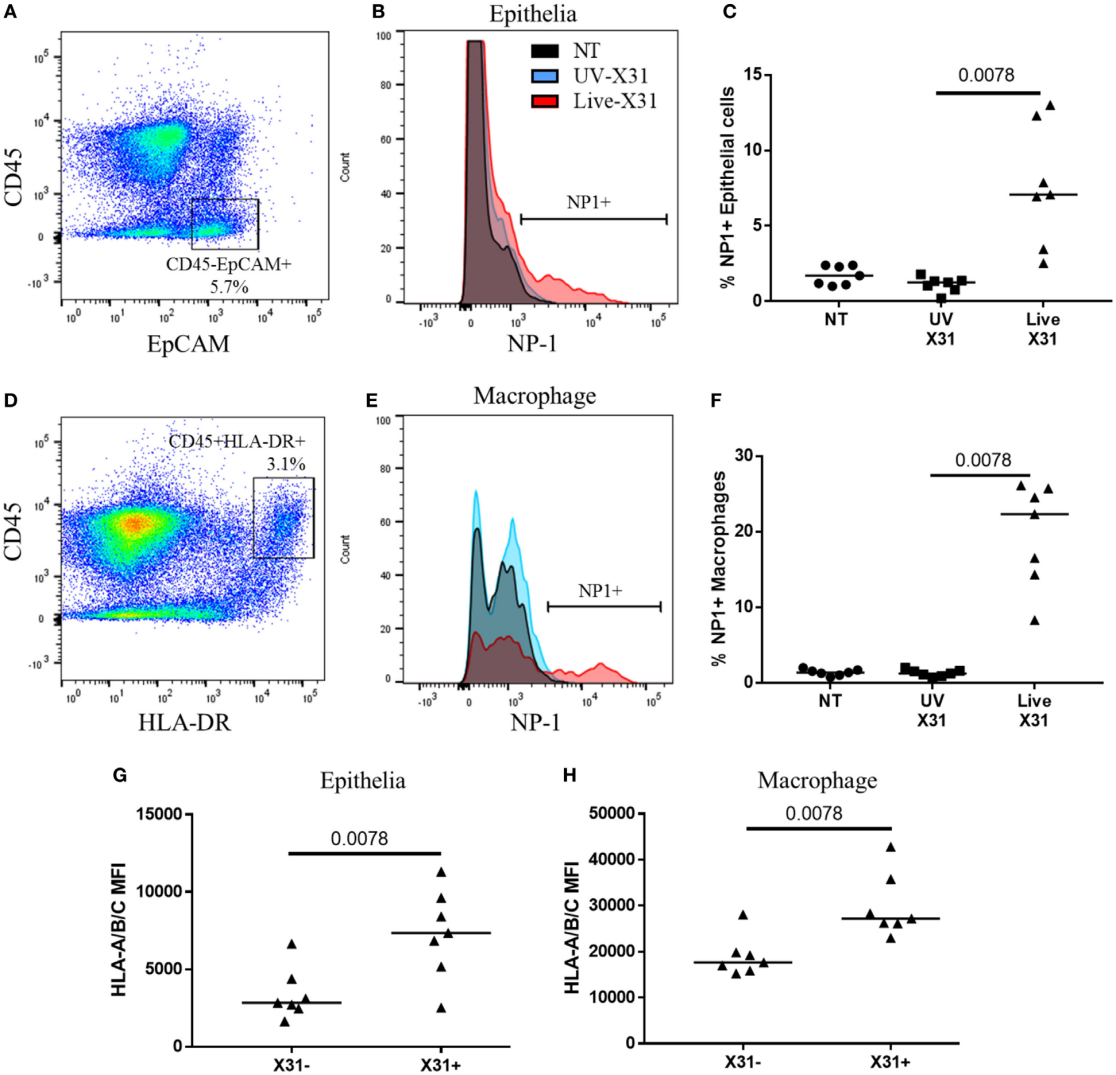
Influenza X31 infection in human lung explants. **(A)** Representative flow cytometry plot defining lung epithelia as CD45^−^EpCAM^+^ cells. **(B,C)** Influenza nucleoprotein-1 (NP1) expression in X31-infected epithelia. **(D)** Representative flow cytometry plot defining lung macrophages as CD45^+^HLA-DR^+^ cells. **(E,F)** Influenza NP1 expression in X31-infected macrophages. **(G,H)** Expression of HLA-A/B/C on epithelial cells **(G)** and macrophages **(H)** with and without X31 infection, as defined by NP1 expression. Lines describe medians, statistical analyses performed by Wilcoxon signed-rank test (*N* = 7).

The co-expression of CD49a, CD69, and CD103 on lung and blood CD56^bright^ NK cells was analyzed as shown in Figures 5A,B and visualized in t-SNE plots (Figures 5C,D). Lung CD56^bright^ NK cells clustered together and appeared distinct to CD56^bright^ of matched peripheral blood (Figure 5C). Analysis of CD49a, CD69, and CD103 expression on lung NK cells suggests that the CD56^bright^ population consists of NK cells that are single-positive (SP), DP, and triple-positive for these markers (Figures 6D,F). Whereas only CD69 could be identified in the peripheral blood CD56^bright^ population (Figures 5B,G and 4E). SP CD49a^+^ NK cells were positioned together with triple- and DP populations within the t-SNE analysis; however, CD56^bright^ NK cells that were SP for CD69 appeared more distinct, as did NK cells negative for all tested residency markers (Figure 5D). CD69 and CD103 were both strongly co-expressed on CD56^bright^CD49a^+^ NK cells, indicating that this population may be resident within the lungs (Figure 5E). One of the largest populations of lung CD56^bright^ NK cells were CD49a^+^CD69^+^CD103^+^, which made up 20.9 ± 12.66% of the CD56^bright^ lung NK cell population (Figures 5E,F).

### *Ex Vivo* IAV (H3N2) Infects the Macrophages and Epithelia of Human Lung Explants

Natural killer cells make up a substantial proportion of the lung CD45^+^ leukocytes (18.55 ± 14.98%) and their response to respiratory infection may have important implications for human disease. Furthermore, lung-resident NK cell populations such as the CD49a^+^ NK cells may be shaped by insult and homeostasis within the lung microenvironment. Therefore, to understand the functional relevance of the lung NK cell phenotypes described here, the response of lung NK cells to IAV infection was characterized in human lung parenchyma. The lung explants were infected with 200,000 pfu/mL UV-irradiated or live X31 IAV for 2 h before removal of extracellular virus and further culture for a further 22 h. Cells were defined as infected when viral NP1 was detected (Figures 6A,B). UV-irradiated IAV was not found to replicate within the lung tissue, as determined by NP1 expression, indicating that the virus is no longer viable (Figures 6C,F). In this model, 7.07% of lung epithelia (defined as CD45^−^EpCAM^+^ cells, Figures 6A,B) and 22.3% of macrophages (defined as CD45^+^HLA-DR^+^ cells, Figures 6D,E) were infected with IAV. Both the airway epithelium and macrophages increased expression of cell surface HLA class I, a key molecule controlling NK cell activation, in response to infection (epithelium *P* = 0.0078, macrophage *P* = 0.0020, Figures 6G–H).

### NK Cells Activate in Response to Influenza Infection of Human Lung Explant

**Figure 7:**
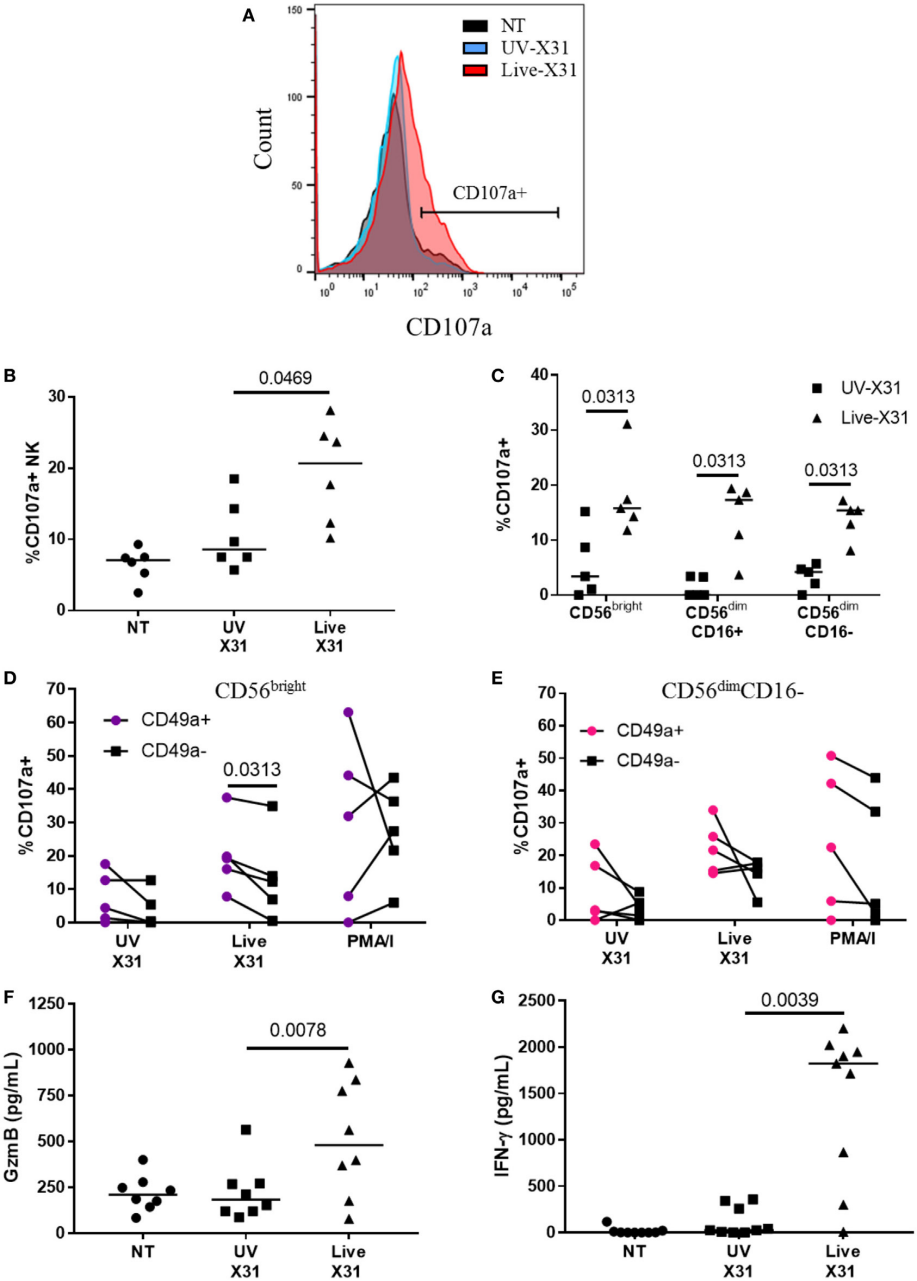
Natural killer (NK) cell activation in X31-infected human lung explants. **(A,B)** Representative flow plot and quantification of surface CD107a on explant NK cells 24 hpi (*N* = 6). **(C)** Surface CD107a on NK cell subsets 24 h after X31 infection (*N* = 5). Uninfected (NT) background CD107a expression was subtracted. **(D,E)** CD49a^+^ and C49a^−^ NK cell degranulation of CD56^bright^ **(D)** and CD56^dim^CD16^−^ **(E)** NK cells following X31 infection and phorbol myristate acetate/ionomycin stimulation of lung explants (*N* = 6). **(F,G)** Extracellular granzyme-B (GzmB) and IFN-γ in explant supernatants 24 h post-infection with X31 (*N* = 8). Lines describe medians, statistical analysis performed by Wilcoxon signed-rank test.

Natural killer cells were strongly activated 24 h post influenza infection (hpi), with a twofold increase in surface CD107a when compared with UV-irradiated X31 treated explant (Figures 7B, *P* = 0.047). NK cell degranulation was not dominated by any particular subpopulation as CD56^bright^, CD56^dim^CD16^+^, and CD56^dim^CD16^−^ NK cells all expressed similar levels of CD107a in X31-infected tissue (Figure 5C). There was a slight trend toward increased NK cell degranulation when MDMs were exposed to UV-irradiated virus compared with uninfected (NT) controls (*P* = 0.094, Figure 7B). Since multiple influenza infections may be experienced throughout life, we hypothesized that prior exposure to influenza may have resulted in increased functionality of the lung CD49a^+^ NK cell populations (Figure 4). To investigate this, the degranulation of CD56^bright^CD49a^+^ NK cells in response to X31 infection was compared with CD56^bright^CD49a^−^ NK cells. As shown in Figure 7D, the degranulation of CD56^bright^CD49a^+^ NK cells was increased relative to CD56^bright^CD49a^−^ NK cells (12.2 vs 19.2%, *P* = 0.031) 24 hpi, indicating that these NK cells may be more responsive to influenza infection. Furthermore, CD56^bright^CD49a^+^ and CD49a^−^ NK cells were not differentially activated when explants were treated with PMA/I, indicating that the total potential of CD56^bright^CD49a^+^ NK cells is similar to CD56^bright^CD49a^−^ NK cells. Thus, this may be a virus-specific activation of CD49a^+^ NK cells. CD49a was also found to be expressed on CD56^dim^CD16^−^ NK cells; however, no difference in the response to virus was observed between CD56^dim^CD16^−^ CD49a^+^ and CD49a^−^ NK cells (Figure 7E).

**Figure 8:**
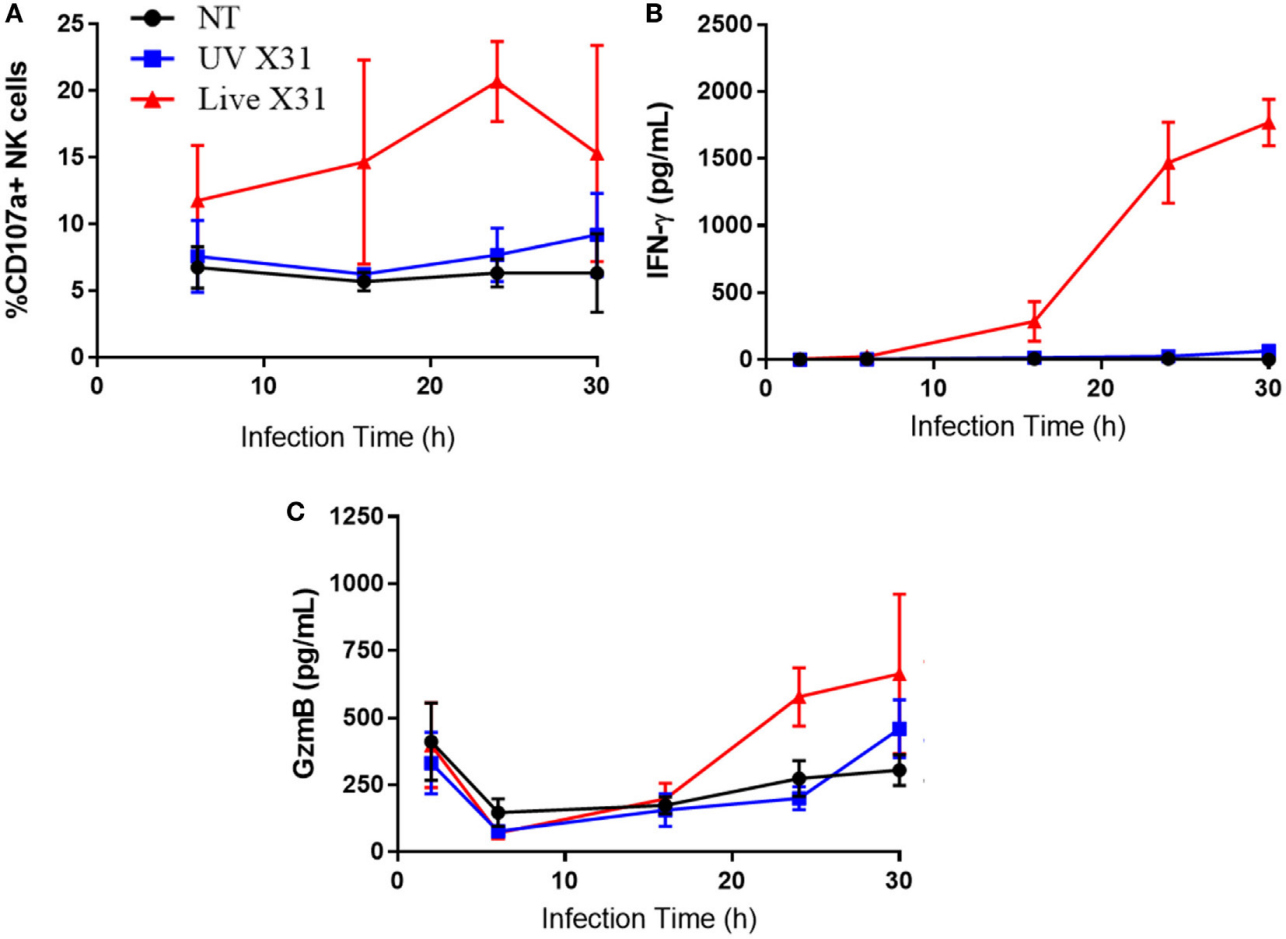
Explant response to X31 infection over time. **(A)** Natural killer (NK) cell surface expression of CD107a. **(B,C)** Extracellular secretion of IFN-γ **(B)** and granzyme-B **(C)** from infected lung tissue (*N* = 3, except 6 h *N* = 2). Lines describe mean and SEM.

Increased CD107a on the NK cells surface was mirrored by increased release of granzyme-B (GzmB) and IFN-γ (Figures 7F,G, *P* = 0.0078 and *P* = 0.0039, respectively). GzmB and IFN-γ are key molecules associated with NK cell activation and were found to rise over a similar time-course with NK cell degranulation (Figure 8). Taken together, the results presented here indicate that lung-associated NK cells activate rapidly as a result of *ex vivo* IAV infection. To further test if this was a discrete effect of NK cells or related to other cell types present in the explant model, we investigated the functional effects of NK cell activation in direct co-culture with infected macrophages.

### NK Cells Are Cytotoxic Toward IAV-Infected MDMs

**Figure 9:**
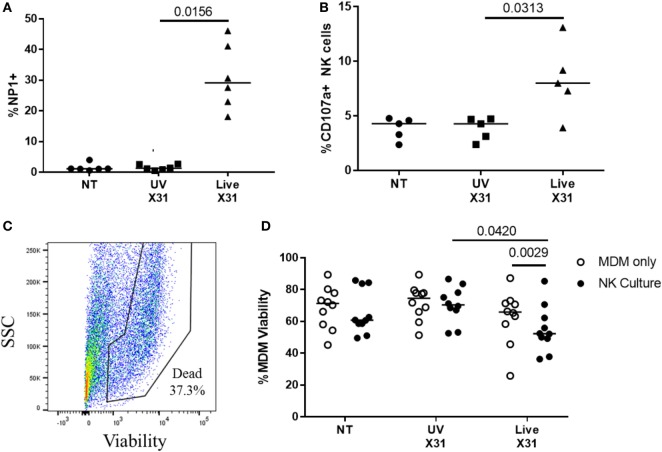
Peripheral blood natural killer (NK) cells are cytotoxic toward X31-infected cells. **(A)** X31 infection of monocyte-derived macrophages (MDMs) was determined by flow cytometric analysis of nucleoprotein-1 (NP1) expression (*N* = 6). **(B)** Uninfected (NT), UV-irradiated X31, and live X31-infected MDMs were cultured with autologous peripheral blood NK cells for 4–6 h. NK cell degranulation was determined by flow cytometric analysis of surface CD107a (*N* = 5). **(C,D)** Following culture with NK cells, MDM viability was measured by uptake of amine-binding dye and analyzed by flow cytometry (*N* = 10). Representative gating for MDM viability. Fixable dead stain, viability gating defined through heat-killed control. **(C)** Representative gating of X31-infected MDM viability. Lines describe medians, statistical analysis by Wilcoxon signed-rank test.

Airway macrophages were a target of H3N2 IAV infection in the explant lung model and have been shown to be critical to influenza control in mice (56–58). To investigate the effects of NK cell activation during IAV infection, we cultured peripheral blood NK cells with IAV-infected autologous MDMs. MDMs were differentiated to a lung-like phenotype, modeling airway macrophage activity and infected as described previously (45), resulting in a median of 29% of MDMs being positive for viral NP1 (Figure 9A). Following infection of the MDM monolayer, purified autologous NK cells were cultured with infected MDMs at an E:T ratio of 1:5 for 4–6 h resulting in increased NK cell degranulation (*P* = 0.031, Figure 9B). To assess NK cell cytotoxicity, MDM viability was measured by flow cytometry (Figures 9C,D). Co-culture with NK cells did not alter MDM viability when MDMs were uninfected (NT) or treated with UV-irradiated X31 (UV-X31) (*P* = 0.2158 and *P* = 0.3848, Figure 9D). Although some variation in the uninfected NK culture was observed, this was not found to be significantly different to either the NT MDMs alone or the UV-X31 exposed co-culture (*P* = 0.2158 and *P* = 0.1250, respectively, Figure 9D). However, when MDMs were infected with live X31, MDM viability was reduced by 12% when NK cells were also present, relative to MDMs alone (*P* = 0.0029, Figure 9D). MDM viability was also reduced relative to UV-X31 treated co-culture and uninfected co-culture (*P* = 0.0420 and *P* = 0.0186, Figure 9D) indicating that NK cells exerted a cytotoxic effect following live X31 infection.

### NK Cell Contact With Infected Cells Determines NK Cell Activation

**Figure 10:**
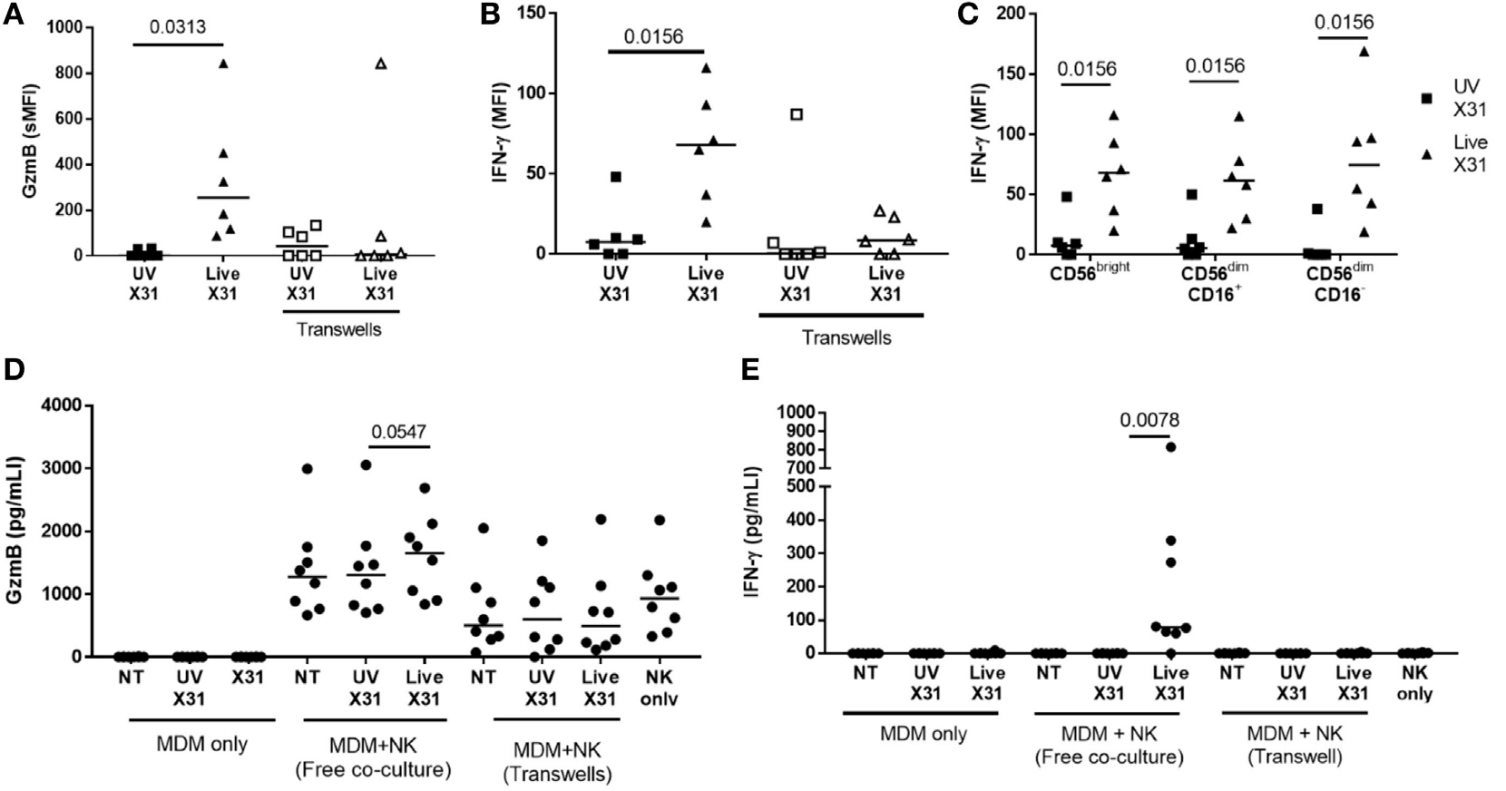
Peripheral blood natural killer (NK) cells produce IFN-γ and granzyme-B (GzmB) after contact with X31-infected cells. **(A,B)** Intracellular accumulation of GzmB and IFN-γ was measured by flow cytometry 6 h after culture with X31-infected monocyte-derived macrophages (MDMs). Physical separation of MDMs and NK cells in a transwell system abrogated IFN-γ and GzmB production (*N* = 6). **(C)** Intracellular IFN-γ of CD56^bright^ and CD56^dim^ NK cell subsets when cultured with MDMs treated with UV-irradiated X31 and live-X31. **(D,E)** Extracellular secretion of GzmB **(D)** and IFN-γ **(E)**. Lines describe medians, statistical analysis by Wilcoxon signed-rank test.

Culture of purified autologous NK cells with IAV-infected MDMs induced NK cell expression of antiviral molecules such as GzmB and IFN-γ, as detected by flow cytometry (Figures 10A,B; Figure S2 in Supplementary Material). Indeed, extracellular IFN-γ was only observed when NK cells were cultured with X31-infected MDMs (*P* = 0.0078, Figure 10D). In addition, there was a non-significant trend toward increased extracellular GzmB in X31-infected co-cultures (*P* = 0.055, Figure 10E). CD56^bright^ and CD56^dim^ NK cells responded equivalently to culture with X31-infected MDMs, with all subsets producing IFN-γ (Figure 10C). These results suggest both cytokines and cytotoxic molecules are released by NK cells following contact with influenza-infected cells.

**Figure 11:**
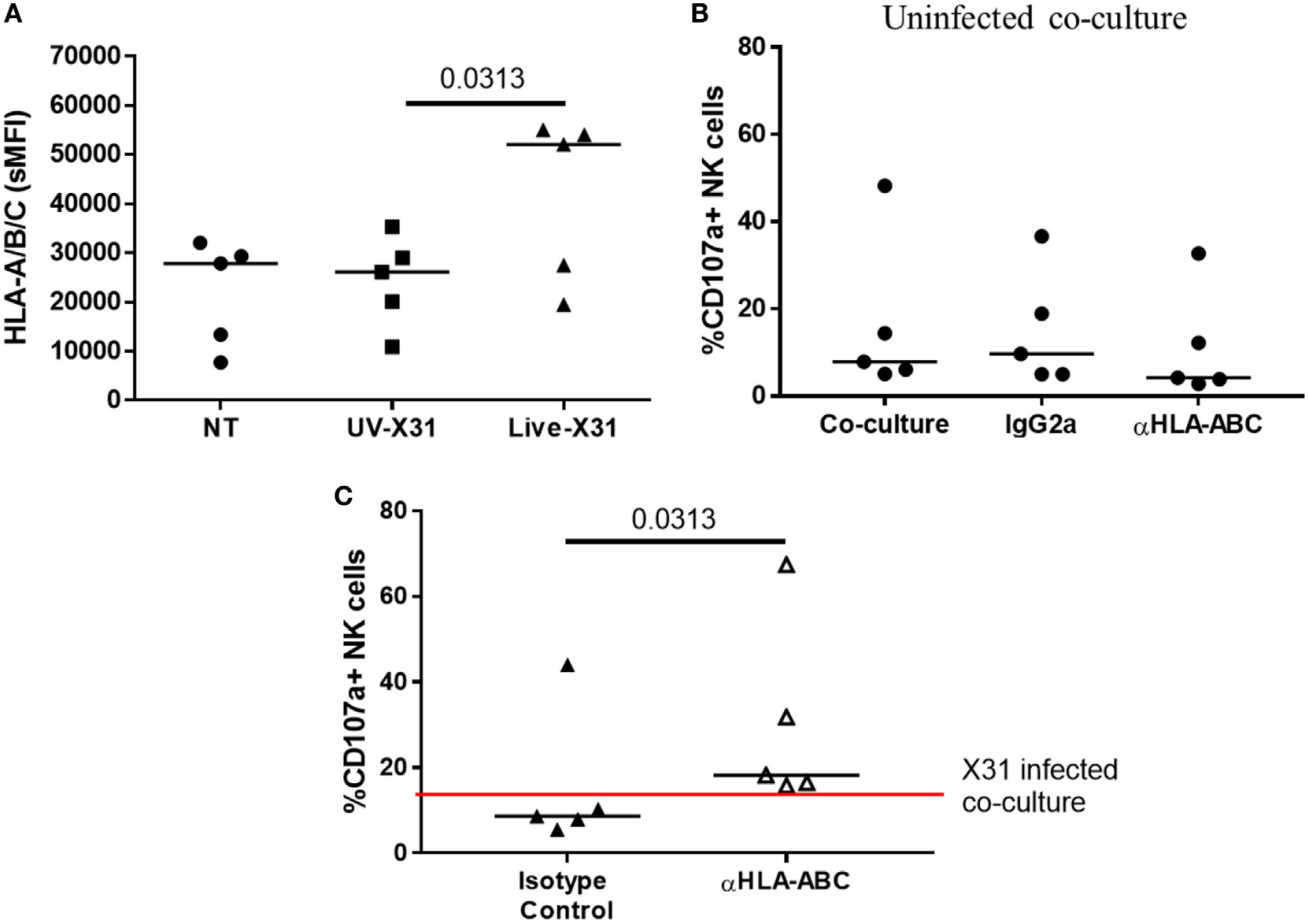
Blocking class I HLA increases natural killer (NK) cell degranulation during culture with X31-infected macrophages. **(A)** Monocyte-derived macrophage (MDM) HLA-A/B/C expression 24 h after uninfected (NT) UV-irradiated and live X31 infection (*N* = 5). **(B)** Background NK cell degranulation following culture with uninfected macrophages when macrophages were treated with αHLA or isotype control for 20 min prior to addition of NK cells (IgG2a) (*N* = 5). **(C)** 24 h post-infection MDMs were incubated with antibody against HLA-A/B/C for 20 min prior to co-culture with NK cells. NK cell degranulation was measured by flow cytometry (*N* = 5). Lines describe medians. Statistical analyses performed by Wilcoxon signed-rank test.

Direct contact between NK cells and MDMs was essential for NK cell activation as separation of the two cells in a transwell system abrogated both GzmB production and IFN-γ release (Figure 10). This suggests that changes to the MDM surface as a result of IAV infection may determine NK cell activation. HLA class I molecules play an important role in governing NK cell responses and are upregulated on IAV-infected lung epithelial cells and macrophages (Figures 6H,I and 11A). To investigate the effect of this change in surface HLA, HLA class I ligands on human MDMs were blocked with anti-HLA-A/B/C (clone W6/32) prior to culture with NK cells. Blocking with αHLA-ABC or isotype control did not affect NK cell activation in response to uninfected MDMs, suggesting that this antibody does not induce antibody-dependent cellular cytotoxicity (Figure 11B). However, during culture with X31-infected MDMs, blocking class I HLA increased NK cell CD107a expression (*P* = 0.031) indicating that HLA class I has inhibitory effects on NK cells during live influenza infection (Figure 11C). Despite this, NK cells still activate in response to influenza-infected cells, suggesting the balance between NK cell activatory and inhibitory signaling is perturbed during contact with IAV-infected cells. These results suggest that NK cells are capable of a strong antiviral response following contact with influenza-infected cells, with production and release of IFN-γ, GzmB, and significant cytotoxicity against infected macrophages.

## Discussion

The role of NK cells in tissue-specific responses is being increasingly recognized as they may represent an important early front-line defense during respiratory infection (32, 35). In this study, we have explored the NK cell response to influenza infection of human lung parenchyma and MDMs. We identified early activation of NK cells in response to influenza-infected cells, including IFN-γ and GzmB production, degranulation, and cytotoxicity. In addition, for the first time, we demonstrate NK cell-mediated destruction of influenza-infected macrophages, indicating that NK cells may have an important role in regulating the effects of antigen-presenting cells during IAV infection (59, 60).

To explore the *ex vivo* function of human lung NK cells, NK cells were defined as CD45^+^CD3^−^CD56^+^ cells, a gating strategy designed to exclude innate lymphoid cell (ILC) populations. Although a small population of ILC3s may be included in our analysis (50% of NCR^−^ ILC3s express CD56) the total human lung ILC3 population comprise less than 0.025% of CD45^+^ cells and therefore would make a minimal contribution to our analysis (61, 62). However, consistent with previous reports, CD56^+^CD3^−^ NK cells made up a significant proportion (18.55%) of CD45^+^ lymphocytes in the human lung and were found to be predominantly mature, canonical NK cells, corroborating the work by Marquardt et al. (37–39).

Understanding the lung NK cell phenotype is important for understanding NK cell function during pulmonary health and disease. The differentiated and active NK cell phenotype described for NK cells both here and by Marquardt et al. (39) is interesting as unchecked cytotoxicity in this setting may have the capacity to impair lung function (39, 63). Indeed, NK cells taken from people with chronic obstructive pulmonary disease (COPD) were found to be more cytotoxic toward airway epithelia, a functional change which appeared intrinsic to the NK cell, indicating an altered activation state in this disease (63). NK cells isolated from the lungs have often been reported as hypofunctional following stimulation with PMA or K562 cell lines, a finding which might reflect important mechanisms of NK cell regulation in the pulmonary environment (36–39). Yet in this study, IAV infection of human lung explants was sufficient to activate NK cell degranulation. Both CD56^bright^ and CD56^dim^ NK cells were found to activate rapidly in response to IAV, indicating that NK cells may aid early virus control within the lung.

As the lungs are a highly vascularized organ, it is possible that the NK cells examined by this study may have been passing through the lungs during circulation (39). Indeed, the phenotype of lung-associated NK cells is more similar to the NK cells of the peripheral blood than other human organs (30, 31, 33, 34). Although we cannot exclude the possibility that the phenotype reported in our study comes from the peripheral blood, the tissue was washed extensively and rested to remove contaminating blood cells prior to analysis. Analysis of the T cell phenotype in lung tissue utilizing this method supports this ability to remove blood lymphocytes, as only memory T cells are isolated from this organ with little to no presence of naïve T cells (64). Moreover, we have identified a novel CD49a^+^CD69^+^CD103^+^ NK cell population from the human lung parenchyma not found in the blood. Interestingly, the lung possessed more CD49a^+^ NK cells compared with reports from the human liver (31).

To investigate the function of human lung CD49a^+^ NK cells, we analyzed the CD107a expression of CD56^bright^CD49a^+^ NK cells (Figure 7). We observed that a greater proportion of CD56^bright^ CD49a^+^ NK cells activate in response to IAV infection compared with CD56^bright^CD49a^−^ NK cells, an effect not seen with PMA/I stimulation. This suggests that CD56^bright^CD49a^+^ NK cells may respond specifically to viral infection as the total potential of CD56^bright^CD49a^+^ NK cells is not dissimilar to CD56^bright^CD49a^−^. Lung CD49a^+^ NK cells may therefore represent a population of resident NK cells that can be induced to express a more robust recall response following prior exposure to common respiratory pathogens, such as IAV. Indeed, liver CD49a^+^ NK cells generated in influenza-infected mice were protective following adoptive transfer and subsequent influenza challenge, although murine lung CD49a^+^ NK cells were not protective in this model (40). These experiments indicate that NK cell memory of influenza infection could exist within the adult human lung, although this requires further corroboration through analysis of transcription factor expression and epigenetic state. If so, the generation of such local mucosal immunity may have important implications for vaccine design, offering a possibility for increasing strain cross-reactivity and effectiveness (35). However, the full functional role of this CD56^bright^CD49a^+^ population and the governing mechanisms remain to be elucidated.

As expected CD56^dim^CD16^−^CD49a^+^ lung NK cells were not found to be more responsive to X31 infection, a finding that fits with the literature as only CD56^bright^ populations have been associated with residency (30, 31, 33, 34). Some authors have suggested that CD56^dim^CD16^−^ NK cells represent contaminating CD3^+^CD56^+^ NKT cells (65, 66). However, as CD3^+^ cells were specifically excluded from our analysis (Figure 1), we consider it more likely that this population represents activated NK cells that have undergone CD56 and CD16 shedding (66–69). Therefore, CD56^dim^CD16^−^CD49a^+^ lung NK cells might represent functionally exhausted CD56^bright^CD49a^+^ cells. Interestingly, CD56^dim^CD16^−^ NK cells were also expanded in the peripheral blood of donors undergoing resection surgery when compared with healthy controls. A finding which may be due to demographic differences between the cohorts. Our preliminary data suggest that age did not affect the proportion of CD56^dim^CD16^−^ NK cells (Figure 2); however, we cannot rule out an effect of disease and smoking status on the NK cell phenotype (39).

Natural killer cell effector molecules IFN-γ and GzmB were produced by influenza-infected explants 24 hpi (Figure 8). The length of time taken in generating both IFN-γ and GzmB by X31-infected tissue could suggest that this is a secondary response to initial inflammatory signaling through IFN-α and IFN-β, as there is some support for this in the literature (70, 71). Interestingly, extracellular GzmB appeared to wane by 6 hpi, only to rise again at 24 hpi in live X31-infected tissue. However, at the 2 h timepoint, extracellular GzmB was greater in all conditions including uninfected (NT) tissue, this suggests that GzmB release at this time-point may be a result of explant removal from the body or tissue preparation, as explants were washed prior to exposure to virus.

Natural killer cell IFN-γ production and release was also measured from co-cultures with X31-infected MDMs and NK cells, an effect that was dependent on contact between the two cell types (Figure 10). Although CD56^bright^ NK cells have been suggested to dominate NK cell cytokine production, both CD56^bright^ and CD56^dim^ NK cells produced IFN-γ when MDMs were infected with live X31, suggesting that there may be a common mechanism of activation between the two subtypes toward influenza (11, 72). This could include the NKp46 or NKG2D activating receptors as well as activating or inhibitory KIR (16–18). However, these molecules are differentially expressed by CD56^bright^ and CD56^dim^ NK cells, and it is possible that NK cell subsets are activated by different mechanisms during influenza infection (12, 50). Interestingly, GzmB release appeared less dependent on contact between NK cells and infected MDMs; however, as GzmB is released within the immune synapse this could limit detection of changes in the amount of this molecule. Although the differences between cells treated with UV-X31 and live X31 have not been thoroughly explored here, no NK cell response was observed toward UV-infected macrophages, either in the tissue explant or MDM infection. This suggests that NK cells are responding to changes on the target cell, in response to the intracellular replication of live influenza.

The upregulation of HLA class I molecules during IAV infection appeared to be inhibitory to NK cells, as blocking HLA class I increased NK cell degranulation. This might represent viral evasion of the NK cell response (73). Alternatively, HLA class I upregulation could also reflect a host strategy for increased antigen presentation to CD8^+^ T cells. An inhibitory role of the HLA class I suggests that NK cell activation of IAV-infected cells may depend on upregulation of concomitant ligands for activating receptors, such as NKp46 and NKG2D on the target cell surface (16–18). Interestingly, cigarette smoke has been shown to prime the NK cell response to viral infection through increased expression of NKG2D ligands in a murine model of COPD (74). Unfortunately, due to flow cytometry channel limits, it was not possible to investigate tissue ligand expression in the experiments presented here.

Cancer resection is one of the few available sources of human lung tissue, but disease state, smoking history, and medication remain confounding factors in the use of this material (39). Furthermore, the heterogeneity between individuals is reflected in the range of NK cell functional responses and marker expression. Despite these caveats, the NK cell phenotype from the human lung is consistent with healthy mice in terms of maturity and differentiation, and functional results were further confirmed in a co-culture model of IAV infection (37, 38). Analysis of human lung-resident NK cell populations would not be possible without human lung tissue and *ex vivo* infection enables the characterization of NK cell function in a relevant context of human disease.

In conclusion, we identify a unique and putative resident CD56^bright^CD49a^+^ lung NK cell population which underwent greater levels of activation during IAV infection. We speculate that CD49a^+^ NK cells could represent a lung-resident population of NK cells trained by the respiratory environment. Further exploration of this NK cell phenotype is required to understand whether such local mucosal immunity could be manipulated to broaden IAV vaccine cross-reactivity and efficacy. As NK cells make up around a quarter of parenchymal lymphocytes and respond rapidly to IAV infection, NK cells may be well placed to provide early and broad innate immunity to IAV infection in humans.

## Ethics Statement

This study was approved by Southampton and South West Hampshire Research Ethics Committees (13/SC/0416 for healthy control group, 09/H0504/109 for resection and blood donors, 15/SC/0528 for age comparison). All participants provided written informed consent in accordance with the Declaration of Helsinki.

## Author Contributions

Data acquisition, analysis, and interpretation were provided by GC, KO, and KS. All the authors contributed to the drafting of manuscript for important intellectual content and manuscript conception and design.

## Conflict of Interest Statement

KS and TW have applied for a patent for the explant infection model (PCT/GB2010/050821 “*Ex Vivo* Modelling of Therapeutic Interventions”). They report funding from GSK Biologicals SA and AstraZeneca outside of the submitted work. GC, KO, and SK have no potential conflict of interest to declare.

## Abbreviations

APC, antigen-presenting cell; COPD, chronic obstructive pulmonary disease; CR-PB, peripheral blood from cancer resection donor; E:T, effector:target; GzmB, granzyme-B; H-PB, healthy peripheral blood; IAV, influenza A virus; ILC, innate lymphoid cell; IQR, interquartile range; MACS, magnetic-activated cell sorting; MDM, monocyte-derived macrophage; MOI, multiplicity of infection; NCR, natural cytotoxicity receptor; NTHi, non-typeable *Haemophilus influenzae*; NP1, nucleoprotein-1; PBMCs, peripheral blood mononuclear cells; PMA/I, phorbol myristate acetate/ionomycin; SMFI, specific mean fluorescence intensity; ssRNA, single-stranded ribonucleic acid; TCR, T cell receptor.
