# Supplementary material for: Human CD49a+ Lung Natural Killer Cell Cytotoxicity in Response to Influenza A Virus
Source: Front Immunol. 2018 Jul 20;9:1671. doi: 10.3389/fimmu.2018.01671 (PMC6062652; doi:10.3389/fimmu.2018.01671)
Supplement: Supplementary file 1 [file table_1.docx]

**Supplementary Material**


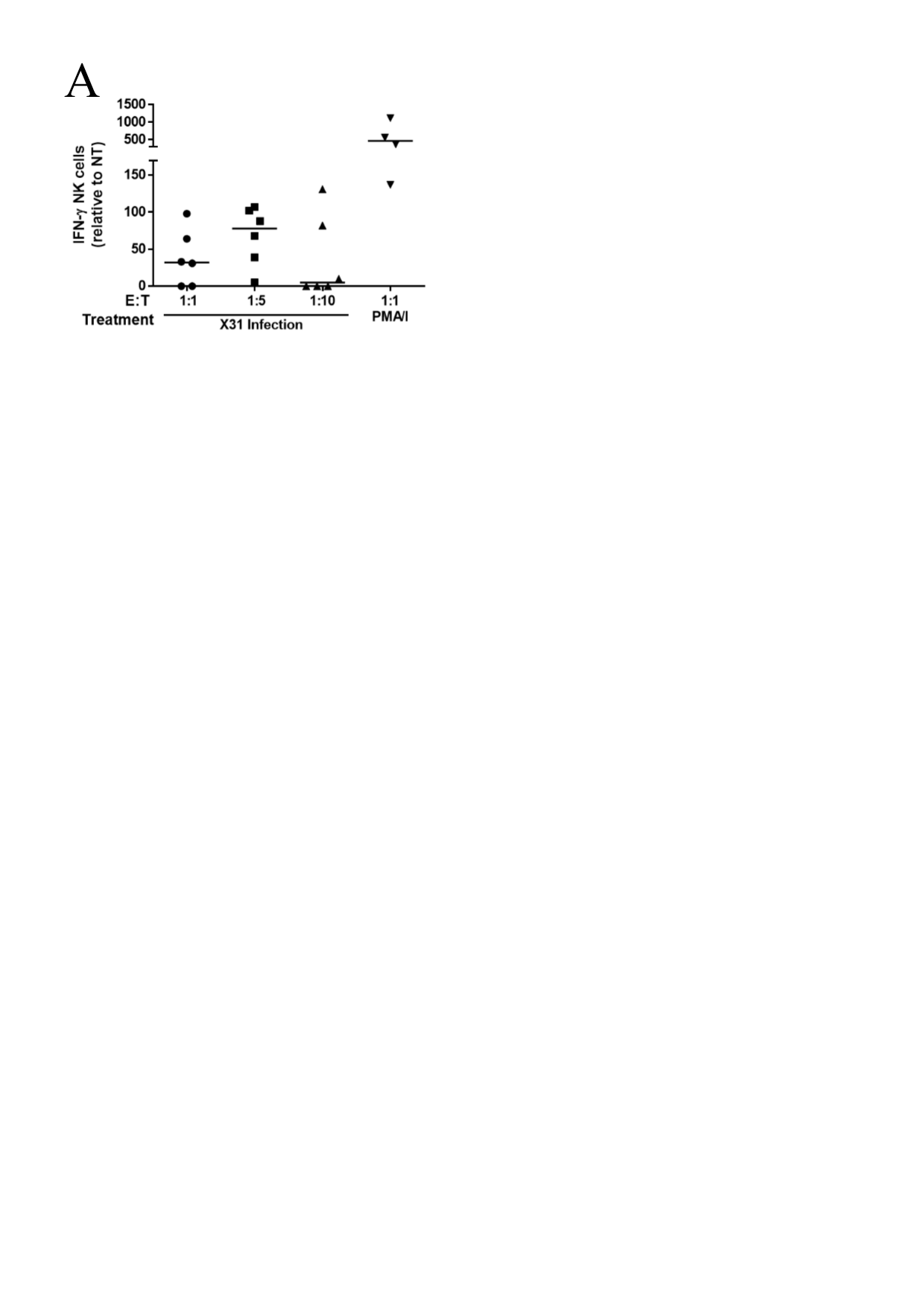


**Supplementary Figure 1**: E:T ratio optimisation for co-culture of peripheral blood NK cells and MDMs. MDMs infected with influenza were washed and then cultured with NK cells for 4h at ratios of 1:1, 1:5 and 1:10 NK cell:MDM respectively. Uninfected co-culture was also stimulated with PMA/Ionomycin as a positive control for NK cell activation. NK cells were assessed for intracellular IFN-γ production by flow cytometry. Lines describe medians.


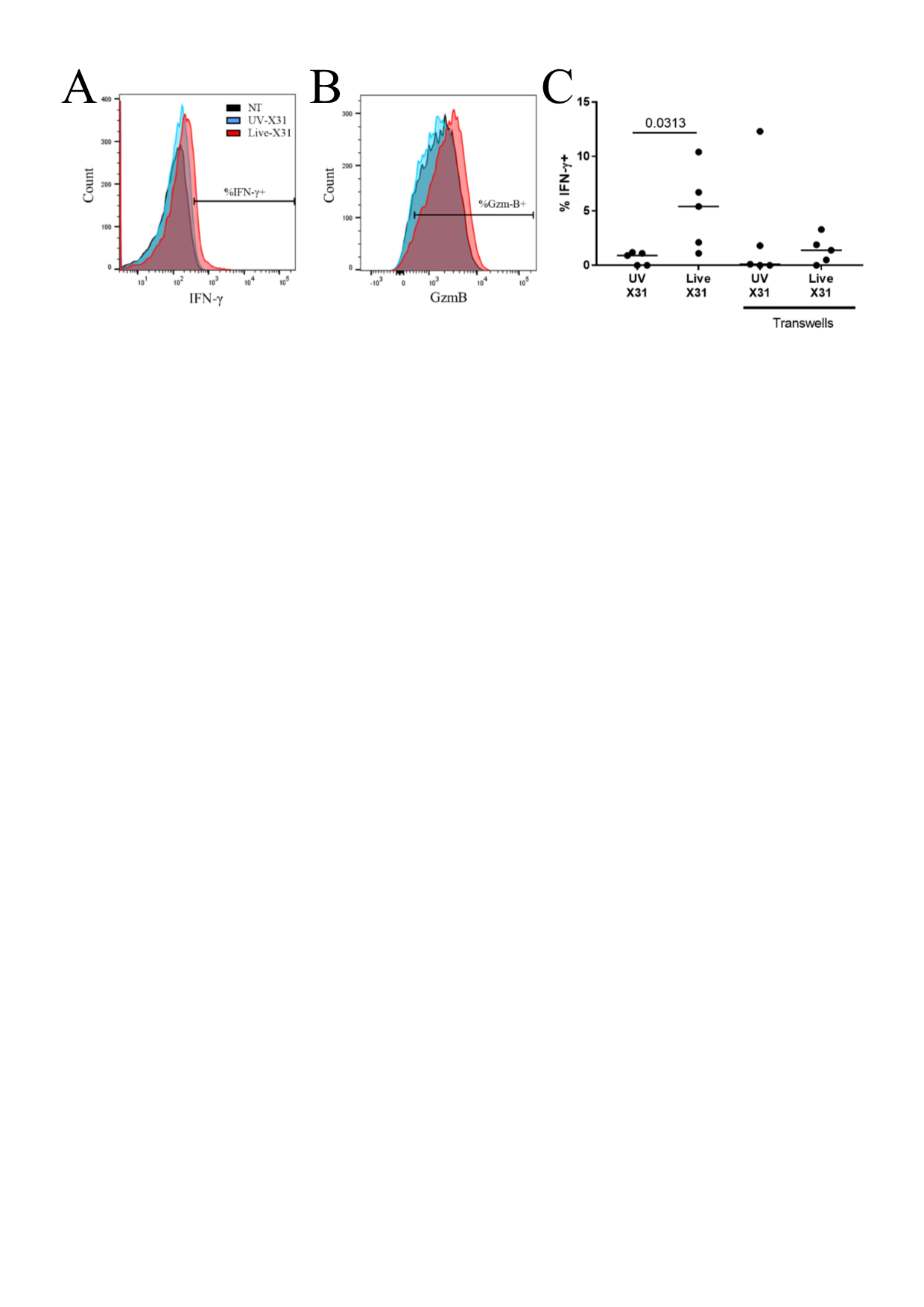


**Supplementary Figure 2:** Representative flow cytometry plots of NK cell activation following culture with X31 infected MDMs. MDMs were not-treated (NT) or treated with UV-irradiated X31 (UV-X31) or live X31 for 24h prior to co-culture. NK cells were cultured with MDMs for 6h at E:T of 1:5 and surface CD107a (**A**), intracellular IFN-γ (**B**) and intracellular Gzm-B (**C**)were measured by flow cytometry. (**D**) Quantification of %IFN-γ+ NK cells following culture with UV-irradiated and live X31 infected MDMs (N=5). %IFN-γ+ from NT control is subtracted from the data. Lines describe medians, statistical analysis by Wilcoxon signed-rank test.
